# Secreted Bacterial Effectors and Host-Produced Eiger/TNF Drive Death in a *Salmonella*-Infected Fruit Fly

**DOI:** 10.1371/journal.pbio.0020418

**Published:** 2004-11-30

**Authors:** Stephanie M Brandt, Marc S Dionne, Ranjiv S Khush, Linh N Pham, Thomas J Vigdal, David S Schneider

**Affiliations:** **1**Department of Microbiology and Immunology, Stanford UniversityStanford, CaliforniaUnited States of America

## Abstract

Death by infection is often as much due to the host's reaction as it is to the direct result of microbial action. Here we identify genes in both the host and microbe that are involved in the pathogenesis of infection and disease in Drosophila melanogaster challenged with Salmonella enterica serovar*typhimurium (S. typhimurium).* We demonstrate that wild-type S. typhimurium causes a lethal systemic infection when injected into the hemocoel of D. melanogaster. Deletion of the gene encoding the secreted bacterial effector *Salmonella leucine-rich (PslrP)* changes an acute and lethal infection to one that is persistent and less deadly. We propose a model in which *Salmonella* secreted effectors stimulate the fly and thus cause an immune response that is damaging both to the bacteria and, subsequently, to the host. In support of this model, we show that mutations in the fly gene *eiger,* a TNF homolog, delay the lethality of *Salmonella* infection. These results suggest that S. typhimurium-infected flies die from a condition that resembles TNF-induced metabolic collapse in vertebrates. This idea provides us with a new model to study shock-like biology in a genetically manipulable host. In addition, it allows us to study the difference in pathways followed by a microbe when producing an acute or persistent infection.

## Abstract 2

A lethal infection of Drosophila is abrogated by removing a secreted Salmonella effector, demonstrating that the fly's immune system, which although initially protective, is subsequently harmful to the host

## Introduction

A hallmark of pathogens is their ability to manipulate their hosts at both a cellular and organismal level. This exploitation of the host can involve the production of specific toxins, secretion of virulence effectors into host cells via type III and type IV secretion apparatuses, or mimicry of host signaling molecules (Young et al. 2002; Gruenheid and Finlay 2003). The host organism can combat the infection with an array of innate and adaptive immune responses. Because there are so many possible combinations of thrusts, feints, and parries, there are many potential outcomes to this battle, ranging from a simple skirmish to defeat for one or the other of the combatants. As a result, microbes can produce acute and virulent infections in diseases such as cholera or plague or can take a path to cause persistent infections as in tuberculosis, Lyme disease, or host-adapted salmonellosis. It is important to understand which bacterial and host factors determine whether a microbe will cause epidemic disease, be a brief nuisance infection, or become a commensal.

Salmonella enterica serovar *typhimurium (S. typhimurium)* is naturally infectious to mice through an oral route and causes a systemic disease resembling human typhoid fever (Lucas and Lee 2000). S. typhimurium crosses the gut epithelium by entering and then killing M cells of the Peyer's patch. Once across the epithelial barrier, *Salmonella* infect macrophages and spread to the mesenteric lymph nodes and subsequently to other organs. S. typhimurium has two type III secretory apparatuses (TTSAs) that translocate effector proteins across both bacterial and host membranes into the host cell cytoplasm. One TTSA encoded by *Salmonella pathogenicity island 1 (SPI1)* plays an important role in cell entry, whereas a second TTSA, SPI2, alters the intracellular environment of the host cell to permit *Salmonella* growth. *Salmonella* likely does not find itself in the hemolymph of *Drosophila* in nature, but by placing it there we can ask some questions that are difficult to approach using other techniques.

The fly is able to fight invading microorganisms via an innate immune response that is composed of at least three arms (Khush and Lemaitre 2000). First, there is an inducible humoral immune response, which involves the secretion of antimicrobial peptides by a liver-like organ called the fat body. Second, there is a melanization response that exposes microbes to reactive oxygen as melanin is deposited on the invader. Third, there is the cellular immune response, which can result in the phagocytosis of relatively small organisms like bacteria or the encapsulation of larger parasites such as nematodes or parasitoid wasp eggs. The humoral immune functions are currently the most thoroughly characterized part of the fly's immune system. This aspect of the immune response is triggered when microbial molecules are recognized by fly pattern recognition receptors and signals are transmitted through the Toll and immune deficiency (IMD) pathways to activate three nuclear factor κB-like transcription factors, dorsal, Dorsal-related immunity factor, and Relish. It was work in this area that led to the discovery of Toll's central role in vertebrate immune recognition (Medzhitov et al. 1997).

Little is known about how *Drosophila* phagocytes affect the course of infections. It has been demonstrated that these cells act in concert with the humoral immune response to destroy invading bacteria (Braun et al. 1998; Elrod-Erickson et al. 2000), and it is well established that they are involved in the encapsulation of parasites (Carton and Nappi 2001). A potential phagocytic receptor for gram-negative bacteria as well as molecules involved in phagocytosis in cultured *Drosophila* cells have been identified (Ramet et al. 2002). Still, we have much to learn about how the phagocytes find and phagocytose microbes, kill microbes, or send signals to the body to indicate that an infection is in progress.

To focus attention on the cellular immune response, we have been characterizing the interaction between *Drosophila* phagocytes and pathogens that are specialized at growing within phagocytes. These bacteria have evolved methods of defeating phagocytes. By using a combination of wild-type and mutant bacteria, we can probe the function of the *Drosophila* phagocyte. We have shown previously that the two intracellular pathogens Mycobacterium marinum and Listeria monocytogenes can infect *Drosophila* phagocytes, and that some of the pathogenesis mechanisms developed by these bacteria for use in vertebrate phagocytes also function in the fly (Dionne et al. 2003; Mansfield et al. 2003). In this work we chose to study S. typhimurium because of its well-characterized secreted effector proteins. We found that the mutation of S. typhimurium effectors led to increased fly survival but, paradoxically, also increased bacterial survival. We propose a model that suggests the immune response of the fly can be deleterious to its health. This model predicts that the fly produces damaging immune effectors. We show that the fly homolog of tumor necrosis factor (TNF), encoded by the *eiger* gene, is involved in this process. Flies homozygous for mutations in *eiger* outlive wild-type flies infected with *Salmonella*.

## Results/Discussion

**Figure 1:**
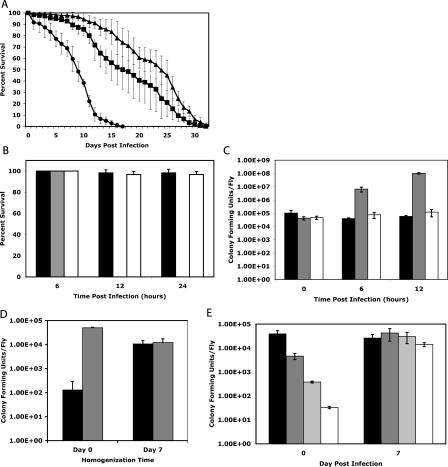
Growth of *Salmonella* in Drosophila melanogaster (A) Survival of wild-type flies injected with *S. typhimurium.* Three sets of 60 flies were injected with approximately 10,000 cfu of SL1344 (Hoiseth and Stocker 1981) from an overnight 37 °C standing culture. Injected flies were incubated at 29 °C. Survival was monitored daily. Circles, S. typhimurium-injected; squares, LB-injected; triangles, uninjected. (B) Survival of immune pathway mutant flies injected with S. typhimurium. Three sets of 20 flies were injected with approximately 100,000 cfu SL1344 and incubated at 29 °C. Fly survival was monitored at 0, 12, and 24 h postinfection. Flies infected: Black, wild-type (Oregon R); gray, *imd^1^/imd^1^;* white,*Dif^1^/Dif^1^*. (C) *Salmonella* growth in infected immunocompromised flies. Flies were injected with approximately 100,000 cfu SL1344 and incubated at 29 °C for the indicated times. Flies were homogenized in LB with 1% Triton X-100 to release bacteria from cells, and the homogenates were plated on LB-streptomycin plates. Only living flies were homogenized. Flies infected: Black, wild-type; gray, *imd^1^/imd^1^*; white, *Dif^1^/Dif^1^*. (D) Effects of gentamicin on S. typhimurium growth in the fly. Wild-type flies were injected with 10,000 cfu of S. typhimurium and then incubated at 29 °C for 7 d. Flies were then injected with either a solution containing 50 nl of 1 mg/ml gentamicin (black) or water (gray). A second group of previously uninfected flies was preinjected with gentamicin or water 15 min before bacterial challenge to determine the effects of the drug on bacteria before they were phagocytosed. All flies were then incubated after gentamicin or water injection at 29 °C for 4 h. Flies were then homogenized and plated. All error bars show standard deviation. (E) Effects of S. typhimurium inoculation size on bacterial growth in the fly. Flies were injected with SL1344 over a 1,000-fold dilution range. Flies were incubated at 29 °C. Flies were homogenized immediately after injection or following 7 d of incubation before plating. Injected concentrations: Black, 0.1 optical density at 600 nm (OD_600_); dark gray, 0.01 OD_600_; light gray, 0.001 OD_600_; white, 0.0001 OD_600_.

We attempted to infect *Drosophila* with S. typhimurium by feeding bacteria to flies and by injecting bacteria into the hemocoel. Flies were resistant to feeding (unpublished data), but direct injection of approximately 10,000 bacterial cells resulted in an infection that caused death in 7–9 d, while injection of sterile medium led to a mean time of death of approximately 20 d (Figure 1A).

The most thoroughly characterized immune response in the fly is the humoral immune response, which involves the secretion of antimicrobial peptides into the hemocoel by an organ called the fat body (Khush and Lemaitre 2000). Two highly conserved signaling systems, the Toll and IMD pathways, have been shown to control the transcription of the antimicrobial peptide genes (Lemaitre et al. 1996, 1997). The IMD pathway is implicated in raising a response to gram-negative bacteria such as *Salmonella*. Flies homozygous for an *imd* mutation succumbed to *Salmonella* infections rapidly, dying within 12 h of infection (Figure 1B). Bacterial numbers exceeded 100,000,000 per fly as the flies died (Figure 1C), and *Salmonella* in dying flies were found circulating in the hemolymph (unpublished data). Circulating bacteria were not seen in wild-type flies. In contrast to *imd* homozygotes, flies homozygous for a mutation in *Dif,* a transcription factor in the Toll pathway, did not die rapidly (Figure 1B and 1C). This experiment shows that mutations in the IMD but not the Toll pathway sensitize the fly to *Salmonella*. Further, these results suggest that the fly's humoral immune response limits the growth of free *Salmonella* in the hemocoel.

In mammals and birds, S. typhimurium is largely an intracellular pathogen. To determine whether S. typhimurium was indeed growing in a protected intracellular niche in the fly, we measured the in vivo sensitivity of *Salmonella* to gentamicin, an antibiotic that does not cross host-cell plasma membranes. To determine whether gentamicin could kill S. typhimurium in flies, 50 nl of 1 mg/ml gentamicin was injected into flies to load them with the drug. S. typhimurium was injected into the flies 15 min later and incubated for 4 h to allow the antibiotic time to act. Flies were homogenized and plated on selective medium to determine *Salmonella* colony forming units (cfu) (Figure 1D). Under these conditions, bacterial loads were 100-fold lower in antibiotic-injected flies than control flies, showing that the antibiotic could kill *Salmonella* immediately following injection. To determine whether S. typhimurium in an established infection were protected from gentamicin killing, we injected flies that had been infected with S. typhimurium for 7 d with gentamicin or water, and incubated the flies for 4 h before homogenizing and plating them. The day 7 time point was chosen throughout this paper, as it is the latest time point we can use before most flies begin to die from the infection. This experiment showed that bacteria that had been in the fly for 7 d were protected from the antibiotic and suggests that the bacteria were located in an intracellular location.

In other bacterial infection models in the fly, death of the host occurs only when bacterial numbers increase beyond 10^6^ bacteria per fly. This is the case for infections both of wild-type flies with pathogens such as Pseudomonas aeruginosa and L. monocytogenes (D'Argenio et al. 2001; Lau et al. 2003; Mansfield et al. 2003) and of mutant flies with nonpathogenic bacteria such as Escherichia coli (Lemaitre et al. 1996; Elrod-Erickson et al. 2000). This is not the case for *Salmonella* in a wild-type fly. To determine the growth characteristics of S. typhimurium in *Drosophila*, bacteria were injected over a 1,000-fold dilution range; 7 d after infection, flies were homogenized and plated. At this time, bacteria were found to cover only a 5-fold range (Figure 1E). Bacteria injected at low densities grew to levels between 10,000 and 100,000 bacteria per fly, but those injected at higher levels did not pass this threshold. This final number is at least 1,000 fold lower than the number of bacteria found during fatal L. monocytogenes and E. coli infections in the fly. *Salmonella* thus reaches a population ceiling in flies, and the flies die containing relatively small numbers of bacteria.

**Figure 2:**
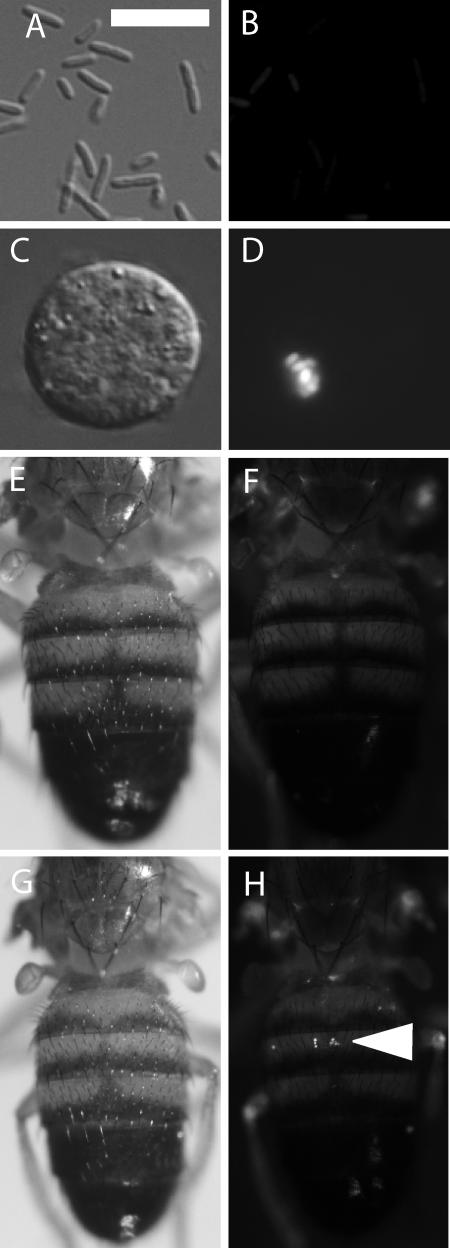
Location of S. typhimurium in the Fly (A–D) Induction of pmig-1 in *Drosophila* hemocytes. SL1344 carrying pmig-1 grown standing at 37 °C overnight were dried to a slide, fixed with formaldehyde and photographed using (A) differential intereference contrast (DIC) optics and (B) GFP optics. The bacteria are not highly fluorescent under these conditions. SL1344 carrying pmig-1 and grown as described above were injected into D. melanogaster larvae and the hemocytes were isolated and fixed after 24 h incubation at 29 °C. A hemocyte is shown in (C) DIC and (D) GFP optics using the same exposures as in A and B. Intensely fluorescent bacteria in the hemocytes are visible. Bar equals 5 um. (E–H) S. typhimurium growth in living flies. SL1344 carrying pmig-1 were injected into wild-type flies and incubated for 2 d at 29 °C. (E and F) Uninfected flies are compared to (G and H) infected flies with (E and G) DIC and (F and H) GFP optics. The arrowhead highlights GFP-expressing *Salmonella* associated with hemocytes on the dorsal side of the fly.

To determine the location of bacteria within the fly, we examined flies infected with S. typhimurium expressing green fluorescent protein (GFP). Bacteria carrying the plasmid containing the macrophage-inducible gene (pmig-1) (Valdivia and Falkow 1997), which induces GFP expression upon entry into the phagosome, or the strain smo22 (Vazquez-Torres et al. 1999), which constitutively expresses GFP (unpublished data), were injected into larvae and flies. GFP-expressing S. typhimurium could be seen within hemocytes bled from infected larvae (Figure 2A–2D). Hemocytes in adults are mostly sessile and cannot be easily removed from the fly. However, these cells can be observed through the cuticle, and clusters of these cells are found on the dorsal surface of the abdomen, along the dorsal vessel (Elrod-Erickson et al. 2000; Dionne et al. 2003). *Salmonella* were found associated with these cells (Figure 2E–2H). The distribution of bacteria seen using the two GFP constructs was similar (unpublished data). This experiment suggests that, similar to the situation in mice, *Salmonella* are found within phagocytes. Furthermore, the *Salmonella* in fly hemocytes induced the expression of GFP from a *Salmonella* promoter normally induced upon phagocytosis by a mouse macrophage. This demonstrates that an S. typhimurium infection-related gene is induced even when the bacteria are infecting an animal at 29 °C instead of 37 °C.

**Figure 3:**
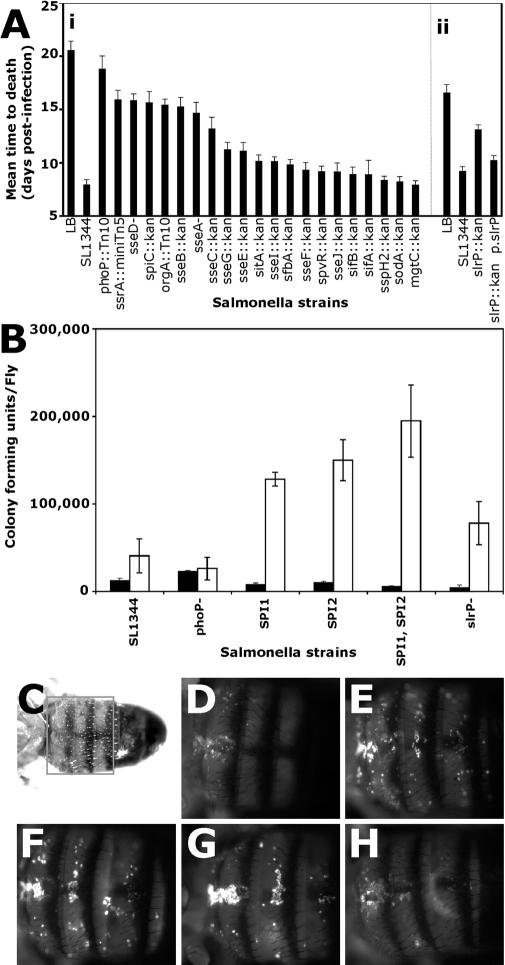
Effects of *Salmonella* Virulence Mutations on Disease in the Fly (A) Mean time to death for infected flies. Identical quantities of S. typhimurium strains were injected into Oregon R flies and survival was monitored daily (i). Infections with *slr*P and rescuing construct were performed separately and are thus reported separately (ii). (B) Growth of mutant *Salmonella* in the fly. Approximately 10,000 cfu of each bacterial strain were injected into flies. Flies were then homogenized and plated at the time of injection (black bars) or following a 7-d incubation (white bars). All error bars show standard deviation. (C–H) Phagocytosis assays in living *Drosophila*. To assay the effects of *Salmonella* infections on phagocyte function, flies were injected with approximately 10,000 cfu of each strain of S. typhimurium. Following a 7-d incubation, the flies were first injected with FITC-labeled dead E. coli and incubated for 60 min to permit them to be phagocytosed. Trypan blue was then injected to quench the fluorescence of extracellular bacteria. The area boxed in (C) was photographed using FITC optics (D–H). The flies were injected with the following bacterial strains: (D) SL1344; (E) LB (control); (F) BJ66 (SPI1); (G) P3F4 (SPI2); (H) *slrP* (Table 1).

S. typhimurium carrying mutations in a gene encoding a regulator of virulence, *phosphatase P* *(phoP)* (Rathman et al. 1996), or blocking the function of either SPI1 (orgA::Tn10) (Jones and Falkow 1994) or SPI2 (ssrA::miniTn5) (Shea et al. 1996), were injected into flies. All of these mutants killed flies more slowly than wild-type bacteria (Figure 3A, graph area “i”). However, analysis of bacterial growth within infected flies revealed a more complicated story. *PhoP* mutants produced infections that were less lethal than wild-type *Salmonella,* but the infecting bacteria grew to the same levels seen in wild-type infections (Figure 3B). In contrast, a *SPI1* or *SPI2* mutant caused little death, and the bacteria grew to an average of 149,000 cfu/fly (*SPI2* mutants) instead of the average of 40,000 cfu per fly found in wild-type infections (Figures 1D and 3B). Thus, the level of bacterial growth of this mutant is almost 4-fold higher than for wild-type *Salmonella*. This suggests that if S. typhimurium cannot manipulate *Drosophila* cells by secreting effectors, the survival of both pathogen and host is dramatically improved.

**Table 1:**
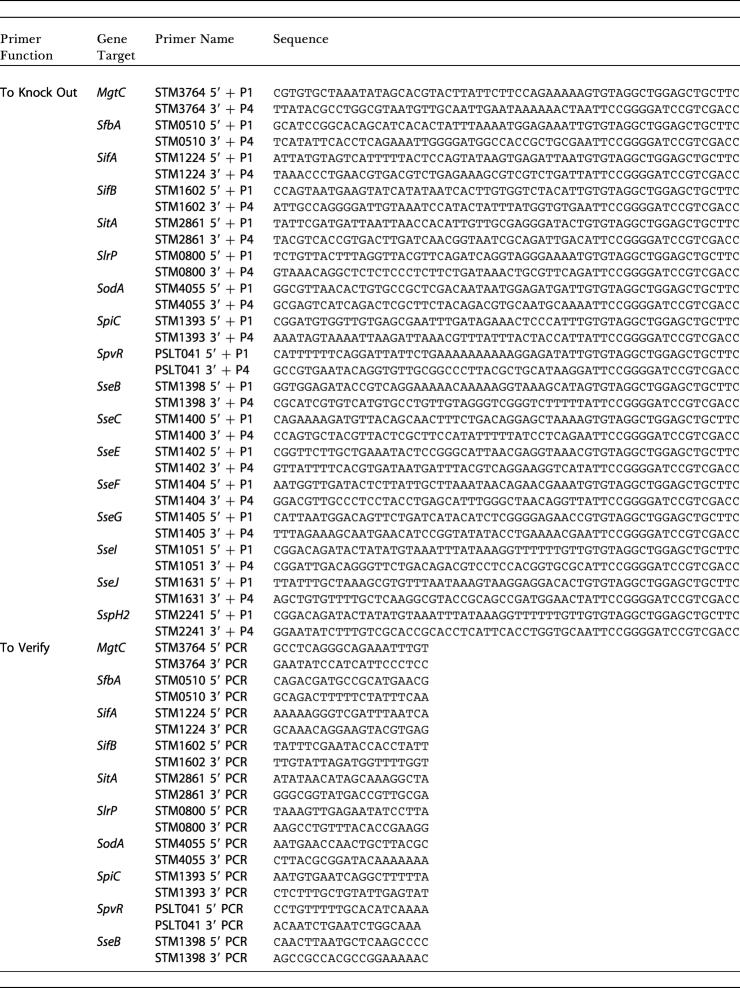
Primers for Mutagenesis and Testing of S. typhimurium

PSLT, plasmid *Salmonella* typhimurium; *Sit, Salmonella iron transport; Sfb, Salmonella ferric binding; Sod, superoxide dismutase; Ssp, Salmonella secreted protein*

**Table 1:**
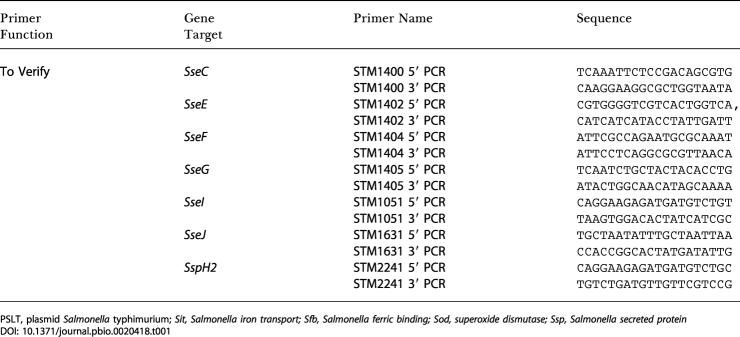
Continued

To determine which of the known TTSA effector proteins contributed to this phenotype, flies were injected with *Salmonella* carrying in-frame deletions of these genes as well as several others we suspected might be involved in virulence in the fly (Figure 3A) (Datsenko and Wanner 2000). Deletions of *spiC, Salmonella secreted effector A (sseA), sseB, sseC,* and *sseD* all resembled a *SPI2* knockout as expected because the proteins encoded by these genes are implicated in building the SPI2 translocation machinery (Nikolaus et al. 2001; Freeman et al. 2002; Ruiz-Albert et al. 2003; Zurawski and Stein 2003). Deletion of the gene encoding the *Salmonella* leucine-rich repeat-containing protein *(PSlrP)* produced a phenotype similar to that of the knockout of the entire *SPI2* TTSA in terms of fly death (Figure 3A, graph area “ii”). Wild-type virulence could be restored to the *slrP* mutant by expressing *slrP,* controlled by its native promoter, *in trans* on a single-copy plasmid, p.*slrP* (Figure 3A, graph area “ii”). Because *SPI1* and *SPI2* bacteria accumulate to higher levels than wild-type strains, in the fruit fly, we measured growth of *slrP-*knockout bacteria. Mutation of *slrP* did not significantly alter the growth of *Salmonella* (Figure 3B). These experiments suggest that *slrP* is a determinant of *Salmonella* virulence in the fruit fly, but that mutation of *slrP* alone is not sufficient to alter *Salmonella* growth. The protein encoded by *slrP* is suspected to be a specific effector translocated directly into the host cytoplasm. Its function there remains unknown (Miao et al. 1999; Miao and Miller 2000; Waterman and Holden 2003).

The results we obtain during fly infection differ from what is found in mammalian and avian hosts. SPI1 is required by *Salmonella* for breaching the gut barrier but is dispensable if the bacteria are introduced systemically by intraperitoneal or intravenous injection. Both *phoP-* and *SPI2*-mutant *Salmonella* are highly attenuated even when injected into mammalian hosts; host survival increases, and the mutant bacteria do not readily replicate and are cleared during infections. In the fly, not only does host survival increase during infection by *SPI1* or *SPI2* mutants but, paradoxically, the bacteria are not cleared and replicate better than their wild-type parents. This is an interesting difference, because it separates mere bacterial presence from pathogenesis and disease. It remains to be determined why the fly does not clear the mutant bacteria in the manner that is seen in the mammalian host.

The analysis of adult hemocytes in the fly is difficult, because we lack good molecular markers for them, and the cells are sessile and rare enough to make them difficult to find regularly in tissue sections. We therefore turned to a functional assay to monitor the behavior of these infected cells by determining their ability to carry out one of their definitive behaviors, phagocytosis. Flies were infected with wild-type and mutant *Salmonella* and then assayed for the phagocytic capacity of the hemocytes on their dorsal surface. Fluorescein isothiocyanate (FITC)-labeled dead E. coli were injected into flies and incubated for 60 min to allow time for phagocytosis to occur. Trypan blue was then injected into the flies to quench the fluorescence of extracellular bacteria. Uptake of the E. coli was then monitored by observing the flies under FITC illumination with a dissecting microscope. At day 1 post-*Salmonella* infection, hemocytes infected with wild-type, *SPI1*-mutant, *SPI2*-mutant, and *SlrP* bacteria showed similar phagocytic activities (unpublished data). Following 7 d of infection, flies infected with wild-type or *slrP*-mutant bacteria had greatly reduced numbers of phagocytic cells (Figure 3C–3H). In contrast, phagocytes remained active during the course of infection by *SPI1* or *SPI2* mutants. Our interpretation of this experiment is that the wild-type *Salmonella* infection results in death of phagocytes or a reduced capacity for phagocytosis. This reduction is not dependent on SlrP function.

**Figure 4:**
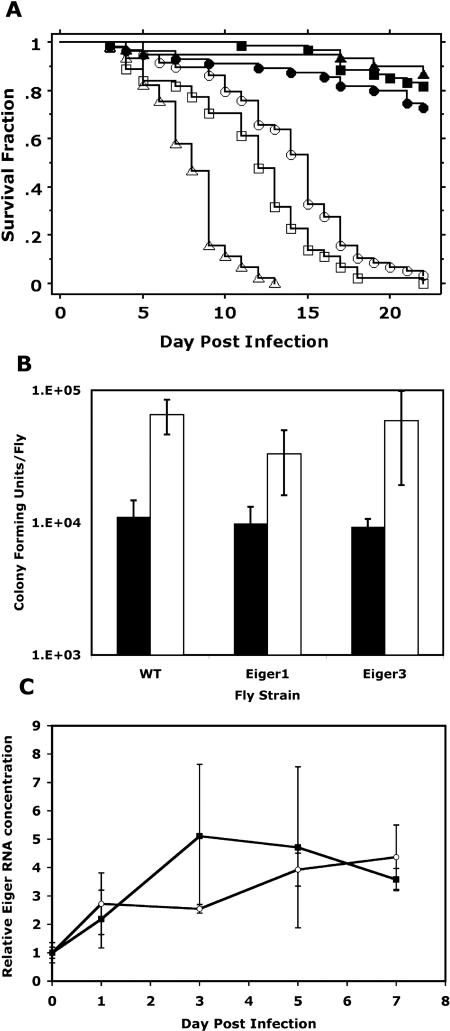
Effects of Eiger Mutations on S. typhimurium Infections in the Fly (A) Survival of *eiger*-mutant flies infected with S. typhimurium. Three sets of 20 flies were infected with 10,000 cfu of SL1344 and incubated at 29 °C. Two *eiger* mutants (*eiger^1^* and *eiger^3^*) and the background strain (*w^118^*) were assayed. Survival was monitored daily. Circle, *eiger^1^/eiger^1^*; square, *eiger^3^/eiger^3^*; triangle, w^118^. Solid shapes indicate LB-injected flies; open shapes indicate SL1344-injected flies. Mantel-Cox analysis demonstrated *p* < 0.001 when comparing infected wild-type to *eiger*-mutant flies. (B) Growth of S. typhimurium in *eiger*-mutant flies. Flies were infected with 10,000 cfu of SL1344 and plated at the time of injection or following a 7-d incubation. Black, time = 0; white, time = 7 d. All error bars show standard deviation. (C) Effects of *Salmonella* infection on *eiger* RNA transcript levels. Total RNA was extracted from five flies per sample on days 0, 1, 3, 5, and 7 postinjection with (open circles) SL1344 or (closed squares) LB. Quantitative real-time RT-PCR was performed. Relative *eiger* transcript quantity is expressed as the fold-difference in comparison to the day 0 value. All error bars show the standard deviation of three RNA preparations.

In mammals, cytokines such as TNF and interferon relay information about infection through the body. Cytokine expression, in particular TNF expression, is responsible directly and indirectly for a significant degree of the pathology observed during microbial infection (Beutler and Rietschel 2003). Flies have one known TNF-like protein, encoded by the gene *eiger* (Igaki et al. 2002; Moreno et al. 2002). Overexpression of *eiger* can induce cell death, but no phenotype had been identified for loss-of-function mutants thus far. We infected wild-type and *eiger^–^* flies with *Salmonella* to determine whether this fly TNF homolog played a role in the pathogenesis of *Salmonella* infections in *Drosophila*. The mean time to death was lengthened by 3 d in the two *eiger* mutants tested (Figure 4A). This experiment suggests that *eiger*/TNF signaling during a *Salmonella* infection contributes to the rate of death of the fly. Accumulation of *Salmonella* did not differ between *eiger* mutants and the background fly strain (Figure 4B). This suggests that *eiger* mutants do not directly affect *Salmonella* growth, but do markedly affect host survival. RNA transcripts of *eiger* were not significantly altered during *Salmonella* infection in comparison to Luria broth (LB)-injected controls (Figure 4C). This suggests that if *eiger* is regulated during *Salmonella* infection, the transcriptional changes are too small to be seen relative to expression in whole flies or the changes are posttranscriptional.

In human disease, morbidity and mortality are often the result of immune responses to invading pathogens rather than to direct action of the pathogens themselves (Beutler and Rietschel 2003). Fever, inflammation, and shock are examples of such processes. In plants, a model is emerging in which host cells monitor important cell functions and respond to their perturbations by pathogens (Staskawicz et al. 2001; Schneider 2002). We suggest the infection caused by *Salmonella* in the fly has attributes of both models. *Salmonella* growth appears to be restricted to hemocytes by action of the humoral immune response. Perhaps the manipulation of these hemocytes by secreted effectors induces additional immune responses that limit bacterial growth. This resembles what is seen in a resistant plant infected by a bacterium expressing the appropriate avirulence protein. Such proteins are secreted into the plant cell's cytoplasm via a TTSA, and they alter the host cell's physiology, presumably to make growth conditions for the bacteria more favorable. Resistant plants can sense this physiological change and raise a type of immune reaction called a hypersensitive response. In the case of *Drosophila,* we suggest that the fly's immune response not only limits the growth of the pathogen but also damages the fly and ultimately leads to its death. When S. typhimurium is prevented from using its secreted effectors, the bacteria survive in the phagocyte and grow to higher numbers, possibly because the bacteria are not being attacked as intensely by the host. The result is that there are more bacteria in the fly and host death is greatly delayed. These two properties appear to be separable. The mutations in *eiger* suggest that there are signaling events that increase the death rate in the fly but do not alter the numbers of *Salmonella*. These experiments provide a new genetic model to explore microbial choice of pathogenic lifestyles in addition to a potential new genetic model for the study of TNF-induced metabolic collapse.

## Materials and Methods

### 

#### Injection assays

Bacteria and medium were injected in a volume of 50 nl through a pulled glass needle. The injection volume was regulated using a Picospritzer III injector (Parker Hannifin, Rohnert Park, California, United States). The needle was placed in the anterior abdomen on the ventrolateral surface. One-week-old male flies were used for all experiments. All experiments were performed in triplicate with at least 20 flies in each replicate. Oregon R flies were used as our wild-type strain.

#### Determination of CFUs in flies

Infected flies were homogenized in LB containing 1% Triton X-100. Three infected flies were homogenized together either with a small pestle or by shaking with 0.4 mm glass beads. Diluted homogenates were plated on LB-agar with 50 μg/ml streptomycin.

#### In vivo phagocytosis assay

This assay was performed essentially as described previously (Elrod-Erickson et al. 2000). Infected flies were injected with 50 nl of 1 mg/ml FITC-labeled dead E. coli (Molecular Probes, Eugene, Oregon, United States) and incubated for 1 h at 25 °C to permit phagocytosis of the bacteria. Trypan blue (4%) was then injected into the flies. Enough dye was injected to turn the entire fly blue. Flies were observed under a Leica MZ3 fluorescent dissecting microscope (Leica, Wetzlar, Germany) using GFP epifluorescence optics, and photographed with an ORCA camera (Hamamatsu, Osaka, Japan) using Openlab software (Improvision, Coventry, UK).

#### Generation of *Salmonella* knockouts and *slrP* complementation plasmid

Isogenic gene knockouts were made in *Salmonella* strain 14028s/pKD46 (Datsenko and Wanner 2000). Transformants were verified by PCR using primers with homology to flanking regions of the target gene. The deleted gene region was transferred to our test strain, SL1344, using standard P22 lambda phage transduction. Transductants were selected on LB agar containing kanamycin and 10 mM EGTA overnight at 37 °C, and mutations were verified by PCR. Primer information is provided in Table 1. Primers designated “Salmonella typhimurium (STM)# 5′ + P1” and “STM# 3′ + P4” were used to generate the kanamycin insert specific to the target gene, using pKD13 as the template. Primers designated “STM# 5′ PCR” and “STM# 3′ PCR” were used to verify recombinants. The bacterial strains and plasmids used for cloning are listed in Table 2. The *slrP* complementation plasmid, p.*slrP* was constructed using the single copy plasmid pDM.2002 (Detweiler et al. 2003). *SlrP* along with its native promoter was amplified from SL1344 genomic DNA using the following primers: 5′- CGCGGATCCAGCGTTGCAGCAGAAAAT-3′ (slrP 5′ BamHI) and 5′- CGCGGATCCTGGGTTAAGCCCGTTTAC-3′ (slrP 3′ BamHI) (Miao and Miller 2000).

**Table 2:**
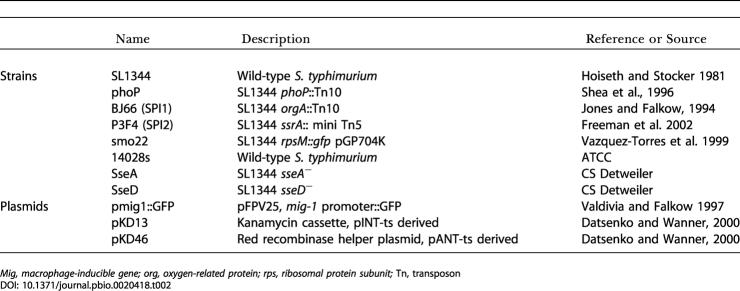
S. typhimurium Strains and Plasmids

*Mig, macrophage-inducible gene; org, oxygen-related protein; rps, ribosomal protein subunit;* Tn, transposon

#### *Eiger* RNA quantitation

Total RNA was extracted from five flies per sample using a Qiagen RNeasy Kit (Qiagen, Valencia, California, United States). Quantitative real-time RT-PCR was performed with rT*th* polymerase (Applied Biosystems, Foster City, California, United States) and the following *eiger* primers: 5′- GATGGTCTGGATTCCATTGC-3′ (5′ oligo), 5′- TAGTCTGCGCCAACATCATC-3′ (3′ oligo) and 5′-6FAM- GACGACGAGGACGACGACGTTAGCT-TAMRA-3′ (hybridization oligo). Concentrations of *eiger* transcripts were normalized to the expression of the D. melanogaster ribosomal protein 15a transcript in each sample (Schneider and Shahabuddin 2000). All experiments were performed in triplicate.

## Abbreviations

cfu: colony-forming unit
DIC: differential intereference contrast
DIF: Dorsal-related immunity factor
FITC: fluorescein isothiocyanate
GFP: green fluorescent protein
IMD: immune deficiency
LB: Luria broth;*mig*
OD_600_: optical density at 600 nm
*pho*: *phosphatase*
pmig: plasmid containing the macrophage-inducible gene
*slr*: *Salmonella leucine-rich*
SPI: *Salmonella* pathogenicity island
*sse*: *Salmonella secreted effector*
STM: *Salmonella typhimurium*
TNF: tumor necrosis factor
TTSA: type III secretory apparatus
